# Consumer-Grade Wearable Sensors for Classifying Pilot Workload and Stress During Real Flight Training: A Leave-One-Subject-Out Validation Study

**DOI:** 10.3390/s26123627

**Published:** 2026-06-06

**Authors:** Rongbing Xu, Shi Cao, Michael Barnett-Cowan, Elizabeth Irving, Ewa Niechwiej-Szwedo, Suzanne Kearns

**Affiliations:** 1 Department of Systems Design Engineering, University of Waterloo, Waterloo, ON N2L 3G1, Canada; 2 Waterloo Institute for Sustainable Aeronautics, University of Waterloo, Waterloo, ON N2L 3G1, Canada; 3 Department of Kinesiology and Health Sciences, University of Waterloo, Waterloo, ON N2L 3G1, Canada; 4 School of Optometry & Vision Science, University of Waterloo, Waterloo, ON N2L 3G1, Canada; 5 Department of Geography and Environmental Management, University of Waterloo, Waterloo, ON N2L 3G1, Canada

**Keywords:** wearable sensors, pilot workload, pilot stress, physiological signals, leave-one-subject-out, real flight, aviation human factors

## Abstract

Consumer-grade wearable sensors may enable continuous monitoring of pilot workload and stress during flight training, yet most prior studies rely on simulators, raw-score labelling, and within-subject validation, limiting generalisability. This study evaluates whether electrodermal activity (EDA), electrocardiogram (ECG)-derived features, and wrist skin temperature, recorded from an Empatica Embrace Plus and a Polar H10 during real Cessna 172 flight training, can classify pilots’ task-relative workload and stress deviations. Thirty-five pilots completed four flight segments and rated workload and stress after each. Fold-safe two-way residual binary labels removed inter-pilot scale-use differences and task-level effects, and five classifiers were evaluated under leave-one-subject-out (LOSO) cross-validation with Benjamini–Hochberg FDR correction. Under LOSO, a Linear SVC on combined features classified stress (macro F1 = 0.607) and XGBoost on EDA classified workload (macro F1 = 0.598) significantly above chance ($p_{\mathrm{adj}}=0.033$); both remained stable under nested cross-validation with an inner hyperparameter search (nested 0.606 and 0.561). A LightGBM model on EDA gave a numerically higher stress score (0.611) that did not survive nested validation. Subject-dependent within-subject validation produced higher apparent performance (macro F1 = 0.853 for stress and 0.791 for workload), but a stricter within-pilot analysis was unstable. These contrasts indicate that personalised classification may be feasible after calibration, whereas uncalibrated cross-pilot prediction in real flight remains modest, with post-flight debriefing the most plausible near-term application.

## 1. Introduction

Pilot workload and stress are recognised determinants of operational performance and aviation safety, and their assessment has long been central to aviation human factors research [1,2,3]. In flight training, workload and stress influence attention allocation, decision-making quality, and the rate at which trainees acquire proficiency. Classical assessment relies on retrospective self-report instruments such as the NASA Task Load Index [4,5] and analogous Likert-type ratings. These instruments offer reasonable face validity but suffer from recency effects, inter-rater scaling heterogeneity, and limited temporal resolution [6,7]. Continuously recorded physiological signals could complement subjective ratings by providing time-resolved, non-intrusive state inference without requiring post-task verbal reports.

A substantial body of autonomic-physiology research supports objective indices derived from electrocardiogram (ECG), electrodermal activity (EDA), and skin temperature signals. ECG-derived metrics, including heart rate variability (HRV) time-domain and frequency-domain features, reflect sympatho-vagal balance and cognitive engagement [8,9,10]. EDA reflects phasic sympathetic arousal via skin conductance responses [11,12]. Peripheral skin temperature captures slower thermoregulatory and vasomotor shifts associated with sustained autonomic activation [13]. However, most evaluations of these signals for workload or stress classification have been conducted in simulators [2,14,15,16], laboratories [17,18], driving paradigms [19], or daily-life monitoring contexts [20,21]. Comparatively few studies have evaluated these signals during real in-aircraft flight, where vibration, motion artefacts, ambient temperature variation, and operational demands pose substantially greater measurement challenges.

Several real-flight neurophysiological studies have demonstrated feasibility. Dehais et al. [22] showed that dry-electrode EEG can detect workload changes during real flight. Taheri Gorji et al. [23] classified pilot cognitive workload from EEG during real flight, and Guo et al. [24] classified pilot fatigue from wearable ECG during actual flight operations. Recent reviews confirm that peripheral-physiological wearable monitoring in operational aviation remains underexplored, with sensor–equipment compatibility and high inter-individual variability identified as key barriers [25,26]. These studies establish that real-flight physiological monitoring is feasible. However, few have evaluated consumer-grade peripheral-physiological sensors for workload and stress classification during real flight using subject-disjoint validation.

Consumer-grade wearables now enable simultaneous recording of ECG, EDA, and skin temperature at low cost and with minimal wearer burden. The Polar H10 chest strap, validated against research-grade ECG for heart rate variability measurement [27,28], provides beat-to-beat R-R intervals from which ECG-derived features can be extracted. The Empatica Embrace Plus wristband records EDA at 4 Hz and continuous wrist skin temperature. These devices are realistic candidates for flight-training deployment because they are commercially available, non-invasive, and compatible with standard flight equipment. However, they introduce signal-quality challenges, particularly motion artefact contamination during active flight manoeuvres, that necessitate careful quality control and cautious interpretation.

Two methodological challenges motivate the analytical framework adopted here. First, most studies use validation designs in which observations from the same participant can appear in both training and test data [17,19,20,22,23]. Subject-dependent validation is appropriate for estimating personalised performance after calibration but can exploit participant-specific physiological signatures rather than learning generalisable state-discriminating features [29,30]. Leave-one-subject-out (LOSO) cross-validation, which holds out all data from one participant at a time, provides a stricter estimate of how well the model would perform for an unseen pilot. Second, most studies classify raw self-report scores (e.g., high vs. low workload based on a median split). Raw-score labels conflate between-pilot scale-use differences with genuine within-pilot state variation and, under fixed task ordering, further absorb task-difficulty effects [29]. Isolating within-pilot deviations from task-level norms focuses the learning problem on individual-level state variation rather than group-level task structure.

This study evaluates whether consumer-grade wearable signals can classify pilots’ task-relative workload and stress during real Cessna 172 flight training. This study makes three distinguishing methodological commitments: (1) physiological data were recorded during actual VFR flight with a certified instructor using the Embrace Plus and Polar H10 [31]; (2) binary labels were constructed from fold-safe two-way residuals that remove both pilot scale-use tendency and task-level mean effects; and (3) the primary evaluation used LOSO cross-validation, with within-subject analyses reported in parallel to distinguish unseen-pilot generalisation from personalised prediction after pilot-specific calibration. We address three research questions: (RQ1) whether consumer-grade wearable physiological signals can classify pilots’ task-relative workload and stress states above chance during real flight under strict LOSO cross-validation; (RQ2) how performance differs between LOSO, subject-dependent within-subject window validation, and stricter within-pilot leave-one-trial-out validation; and (RQ3) which signal modality and classifier combinations are most effective for workload versus stress classification.

## 2. Materials and Methods

This study follows a six-stage pipeline. Physiological signals were first acquired in flight from a chest-strap electrocardiogram and a wristband recording electrodermal activity and skin temperature. Signals were then preprocessed and quality-controlled, segmented into overlapping windows, and converted into modality-specific feature sets. Fold-safe two-way residual binary labels were constructed within each cross-validation fold to remove inter-pilot scale-use and task-level effects. Five classifiers were then trained and evaluated under leave-one-subject-out (LOSO) cross-validation, with within-subject analyses and a full classifier-by-modality factorial reported in parallel. The remainder of this section details each stage in turn.

**Figure 1:**
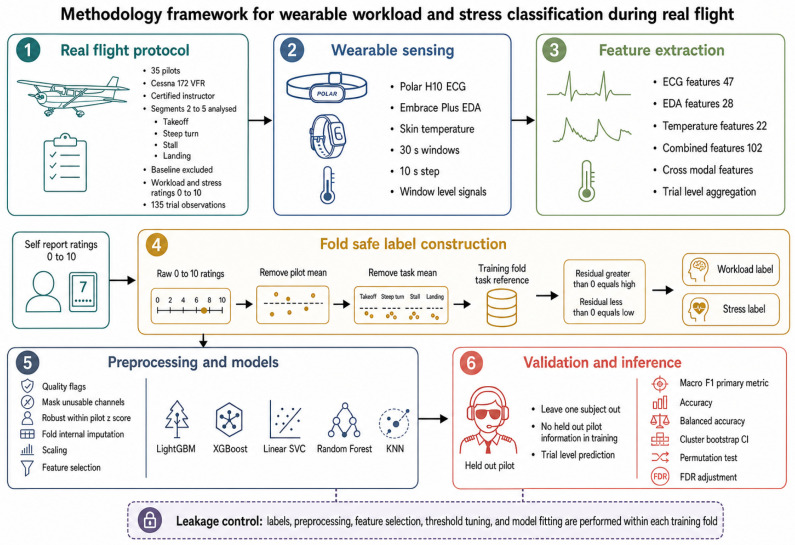
Overview of the analytical pipeline of six stages, from flight experiment through validation output, highlighting the fold-safe two-way residual label construction and leave-one-subject-out (LOSO) cross-validation design.

Figure 1 provides an overview of the analytical pipeline, from data collection through model evaluation.

### 2.1. Participants and Protocol

**Figure 2:**
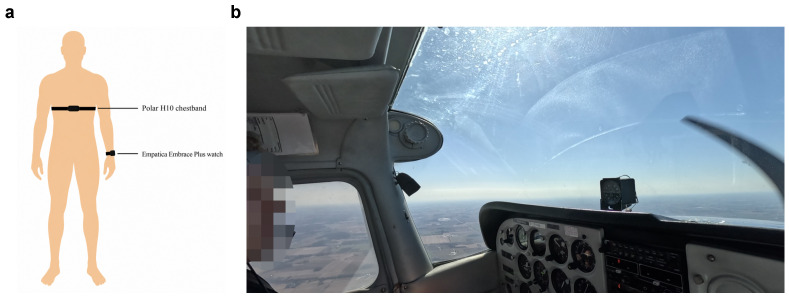
In-cabin experimental setup. (**a**) Placement of the Polar H10 chest strap (ECG) and the Empatica Embrace Plus wristband (EDA and skin temperature). (**b**) Photograph taken during data collection in the Cessna 172 cockpit; the participant’s face is obscured to protect privacy.

**Figure 3:**
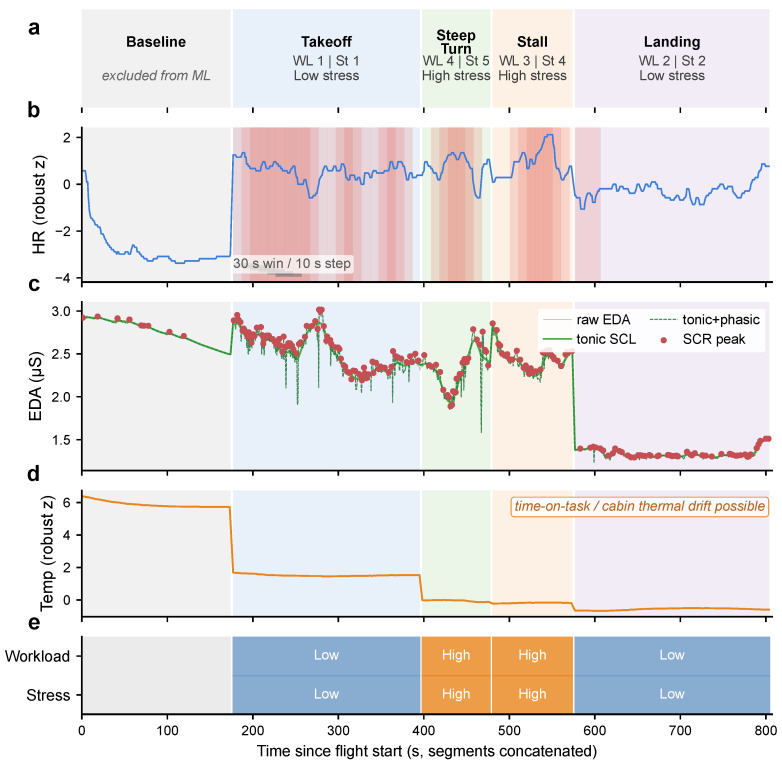
Representative real-flight physiological timeline for one pilot (all models were fit on all pilots and trials). (**a**) Flight-segment timeline with per segment raw workload/stress ratings and residual labels; the background shading distinguishes the successive flight segments (grey Baseline, blue Takeoff, green Steep Turn, orange Stall, purple Landing); Baseline is excluded from the machine learning analysis. (**b**) Normalised heart rate with ECG-unusable windows shaded red and the 30 s/10 s windowing indicated. (**c**) EDA decomposed into tonic SCL and phasic SCR (NeuroKit2 cvxEDA); red points mark SCR peaks (scipy find_peaks, prominence 0.01 μS, minimum spacing 1 s). (**d**) Wrist skin temperature (robust *z*), which drifts slowly across the flight. (**e**) Workload and stress residual label strips. Signals smoothed only by a 5-sample rolling median; segments concatenated back-to-back.

Thirty-five pilots (mixed PPL/CPL/SPL/SPP licences) were recruited from southern Ontario flight-training units. Full recruitment, ethics approval (University of Waterloo ORE #45974), sensor placement, and operational details are reported in [31]. The chest-strap and wristband placement and the in-cabin recording environment are shown in Figure 2, and a representative single-flight physiological timeline with its segment structure and residual labels is shown in Figure 3. Each pilot flew a Cessna 172 under VFR with a certified flight instructor and completed five sequential segments in fixed order: (1) Baseline (ground, 151 s mean), (2) Normal Takeoff (95 s), (3) Steep Turn at $45^{\circ }$ bank (56 s), (4) Power-on Stall (76 s), and (5) Normal Landing (158 s). Baseline was excluded from the machine learning analysis because all pilots rated Baseline workload as 0. After each segment, pilots provided 0–10 Likert-type ratings for workload and stress (verbal anchors at 0, 5, 10) [5]. Single-item ratings were a practical concession to the operational constraints of real-flight data collection and impose a known reliability ceiling [6,7]. Single-item self-reports typically show test–retest reliability of about ICC = 0.75 [32], which sets an upper bound on achievable agreement; the macro F1 values below should be read against this attenuated ceiling rather than against an error-free target. After signal-quality screening, 135 trial-level observations remained available for analysis (Table 1). Participant characteristics are summarised in Table 2. A single pre-flight stress rating was obtained from each pilot; caffeine and nicotine intake, sleep, and medication were not systematically recorded under this operational protocol, and this is noted as a limitation (Section 4.6).

**Figure 4:**
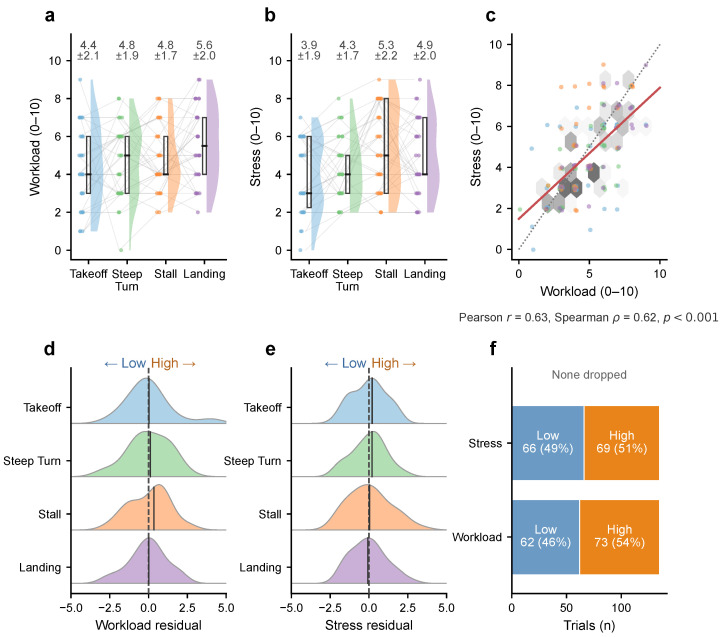
Self-reported workload and stress landscape across real-flight manoeuvres. (**a**,**b**) Raincloud distributions of raw workload and stress by segment: half-violin density, interquartile box, individual pilot points, and faint within-pilot connecting lines; mean ± SD annotated. (**c**) Workload–stress coupling (hexbin density, points coloured by segment throughout (Takeoff blue, Steep Turn green, Stall orange, Landing purple), dotted identity line, red linear fit; Pearson r=0.63, Spearman ρ=0.62, p<0.001). (**d**,**e**) Two-way residual distributions by segment, centred on zero (high to the right, low to the left). (**f**) Resulting two-way residual binary label balance.

Workload scores (segments 2–5) ranged from 0 to 9 (mean =4.89, SD =1.93), with Landing eliciting the highest mean workload (5.57). Stress scores ranged from 0 to 9 (mean =4.62, SD =1.97), with Stall eliciting the highest mean stress (5.26). Workload and stress were moderately correlated (Pearson r=0.626, Spearman ρ=0.624, both p<0.001; Figure 4c), consistent with the expectation that the two constructs are related but not identical. One-way $\eta^{2}$ for the association between segment identity and raw score was 0.049 for workload and 0.068 for stress, indicating modest segment effects after excluding the Baseline floor (Figure 4a,b).

### 2.2. Sensors and Feature Extraction

Physiological signals were recorded from the Polar H10 chest strap (ECG) and the Empatica Embrace Plus wristband (EDA at 4 Hz; skin temperature). All signals were windowed at 30 s with 10 s step (≈two-thirds overlap); window-level features were aggregated to the trial level using a model-specific method (Section 2.5).

ECG-derived features (47) were extracted from R-R intervals and span time-domain (mean RR, SDNN, RMSSD, pNN50, mean HR), frequency-domain (LF, HF, LF/HF), nonlinear (sample entropy, Poincaré SD1/SD2, DFA $\alpha_{1}$/$\alpha_{2}$), respiratory, and engineered ratio categories. Multi-scale variants were computed at 5, 10, and 20 s sub-windows. Beat-to-beat motion artefacts were identified using an automated quality flag. Steep Turn (56 s) and Stall (76 s) segments fall below the five-minute window recommended by the Task Force [8]; frequency-domain features from these segments are interpreted as ultra-short proxies [33,34]. For these durations, the frequency-domain features had near-zero between-segment reliability (e.g., LF/HF intraclass correlation = 0.007), whereas time-domain features were far more stable, consistent with ultra-short HRV studies that find LF/HF invalid below roughly three minutes [35,36,37]. We therefore treat frequency-domain features as exploratory and de-emphasise them; removing them entirely changes ECG performance by at most 0.07 macro F1 (Section 3.3).

EDA features (28) were extracted from Embrace Plus skin-conductance traces decomposed into tonic SCL and phasic SCR components [11,12,38]. Features include tonic SCL statistics (slope, variance, terminal change), phasic SCR descriptors (count, rate per minute, mean and maximum amplitude, mean rise time, mean half-recovery time, AUC), engineered ratios (SCR AUC per SCL), and multi-scale slope estimates at 5, 10, and 20 s sub-windows. The 4 Hz rate is native to this device class (as for the Empatica E4), so a higher-rate reference for decimation is unavailable. The two shape-dependent features (SCR rise time and half-recovery time) are near the 4 Hz resolution limit and are treated as low-confidence; removing them leaves performance unchanged (Section 3.3), so the results rest on 4 Hz robust amplitude, count, and tonic-level features.

Temperature features (22) summarised wrist skin temperature traces via base statistics (mean, slope, variance), an engineered slope feature (°C s^−1^), and multi-scale descriptors. The combined feature set (102) merges all three modalities with cross-modal engineered features (HR-EDA Spearman correlation, peak cross-correlation lag, HR-EDA coactivation fraction, HR-temperature and EDA-temperature correlations). Trials with missing modalities were retained via fold-internal median imputation provided that at least one primary channel was usable.

### 2.3. Label Construction: Fold-Safe Two-Way Residual Binary Labels

Rather than dichotomising raw self-report scores, we constructed binary classification labels using a fold-safe two-way residual approach that removes both inter-pilot scale-use differences and task-level mean effects. For each LOSO fold in which pilot *i* is held out, the training-set grand mean ${\bar{r}}_{\mathrm{train}}$, each training pilot’s mean score ${\bar{r}}_{p}$, and each task’s reference mean ${\bar{r}}_{t}^{\left(-i\right)}$ are computed. Two-way residuals are then:

$${\mathrm{residual}}_{it}=r_{it}-{\bar{r}}_{i}-{\bar{r}}_{t}^{\left(-i\right)}+{\bar{r}}_{\mathrm{train}}$$

where $r_{it}$ is the raw 0–10 rating, ${\bar{r}}_{i}$ is pilot *i*’s mean score across all tasks, and ${\bar{r}}_{t}^{\left(-i\right)}$ is the task reference mean from the training set. Observations with ${\mathrm{residual}}_{it}>0$ are labelled “high” (class 1, the pilot responded more strongly than expected for this task); observations with ${\mathrm{residual}}_{it}<0$ are “low” (class 0). Observations with exactly zero residual are dropped.

This procedure has two important properties. First, because the task reference mean ${\bar{r}}_{t}^{\left(-i\right)}$ is computed exclusively from training pilots, no test-fold label information leaks into the training-label construction (“fold-safe”). Second, by removing both the pilot mean and the task mean, the residual isolates within-pilot, within-task state variation, the component most relevant to detecting whether a particular pilot experienced higher-than-expected workload or stress on a given task.

**Figure 5:**
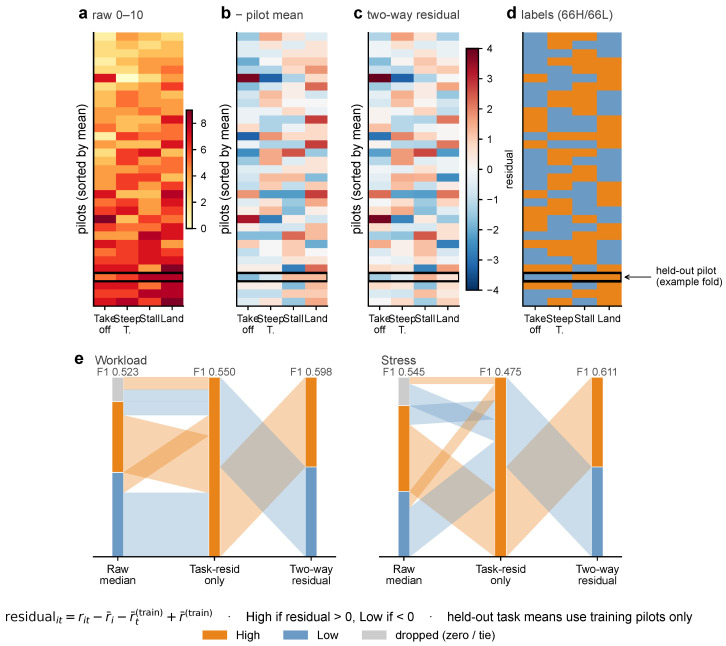
Fold-safe two-way residual label construction (workload shown; one example held-out pilot outlined). (**a**) Raw 0–10 rating matrix (pilots sorted by mean rating). (**b**) Pilot-centred matrix (raw minus pilot mean). (**c**) Two-way residual matrix, with task reference means computed from training pilots only. (**d**) Binary high/low labels. (**e**) Label-switching alluvial from raw median split, through task-residual-only, to the two-way residual labels, for workload and stress, annotated with the corresponding LOSO macro F1. The held-out pilot’s task reference means use training pilots only; test labels are retrospective.

An important clarification concerns the held-out pilot’s labels. For held-out pilot *i*, the pilot mean ${\bar{r}}_{i}$ is computed from that pilot’s complete set of self-report ratings across all four tasks, rendering the test labels retrospective: they require the pilot’s full set of post-flight ratings and are therefore applicable to offline post-flight analysis (e.g., post-flight debriefing) rather than real-time or first-task deployment. The fold-safe property pertains specifically to the training-label construction and the task reference means, which exclude the held-out pilot; the test labels themselves depend on the held-out pilot’s own complete self-report vector (Figure 5). Because each held-out pilot’s own mean is subtracted, that pilot’s four residual labels are approximately zero-sum, limiting within-pilot label balance. This does not bias the aggregate metric, which pools macro F1 at the trial level across all 35 pilots, but it does reduce within-pilot separability. This pattern is consistent with the unstable within-pilot leave-one-trial-out results reported below.

### 2.4. Preprocessing and Quality Control

Synchronisation and annotation were handled as follows. The two devices each carry their own timestamped clock; recordings were aligned to a common timeline anchored to the flight-event markers logged during data collection, and segment boundaries (Baseline, Takeoff, Steep Turn, Stall, Landing) were annotated against these markers. Because segments are analysed at the trial level after window aggregation, sub-second residual clock offsets between devices do not affect the labels. Self-report ratings were associated with their corresponding segment by event marker. Missing or corrupted data were handled by the modality-specific quality flags described below (masking of unusable channels and fold-internal median imputation), and per segment usability rates are reported. Full acquisition and instrumentation details are given in the companion data-collection paper [31].

Preprocessing comprised seven steps: (1) excluding Baseline; (2) retaining only trials with non-missing ratings; (3) applying modality-specific signal-quality flags (combined-modality windows required at least one usable channel); (4) masking features from unusable channels; (5) dropping near-zero-variance columns; (6) performing within-participant robust z-score normalisation (z=(x−median)/(1.4826×MAD+ϵ)) to remove between-participant baseline differences; and (7) performing fold-internal median imputation followed by standard scaling. This normalisation is session-retrospective, suitable for post-flight analysis but requiring adaptation for real-time use.

EDA and temperature were 100% usable across all 1010 windows. ECG was 91.2% usable overall (lowest during Takeoff, 87.1%); 135 of 140 trials (96.4%) had at least one usable ECG window. Trials with missing ECG were retained via fold-internal imputation.

### 2.5. Classification Models

Five classifiers spanning different model families were selected to provide a broad assessment of the classification problem:

1. LightGBM [39] on EDA features (28 features): 400 estimators, 7 leaves, learning rate 0.03, min child samples 8, balanced class weights. Feature selection: top 10 by ANOVA F-test. Window-to-trial aggregation: median. Threshold tuning: training-trial macro F1.
2. XGBoost [40] on EDA features (28 features): 400 estimators, max depth 2, learning rate 0.03. Feature selection: top 20 by ANOVA F-test. Aggregation: trimmed mean. Default threshold (0.5).
3. Linear SVC [41] on combined features (102 features): C=1.0, balanced class weights. Feature selection: ExtraTrees-based SelectFromModel. Aggregation: mean. Threshold tuning: training-trial macro F1.
4. Random Forest [42] on ECG-derived features (47 features): 600 trees, max depth 5, min leaf size 3, balanced subsample class weights. Feature selection: top 20 by ANOVA F-test. Aggregation: median. Threshold tuning: training-window accuracy.
5. K-Nearest Neighbours (KNN) [43] on combined features (102 features): k=7, distance weighting. Feature selection: ExtraTrees-based SelectFromModel. Aggregation: mean. Threshold tuning: training-trial accuracy.

Each model was wrapped in a scikit-learn [44] pipeline (imputation → scaling → feature selection → classifier). Window-level probabilities were aggregated to trial-level predictions via model-specific methods (mean, median, or trimmed mean).

All five configurations were selected a priori; no formal development split was used, and all hyperparameters were fixed before the final LOSO evaluation. Gradient-boosted models were paired with EDA based on prior work [17,19]; Random Forest was paired with ECG for its robustness to collinear HRV features; and SVC/KNN were paired with combined features to test multimodal fusion. A full factorial analysis (Section 3.3) crosses all classifiers with all modalities. To verify that the fixed hyperparameters (for example, k = 7 for KNN) were not responsible for the reported performance, we additionally ran nested LOSO cross-validation with an inner subject-grouped hyperparameter search (Section 3.4); the conclusions were robust.

We deliberately used compact, interpretable classifiers rather than deep sequence models such as LSTMs or Transformers. Under LOSO the effective sample size is the number of subjects (approximately 35), because windows from the same pilot are not independent. This is too few to constrain such architectures without overfitting. Hybrid deep models nonetheless remain promising for naturalistic pilot-stress recognition in larger within-subject settings [45].

### 2.6. Cross-Validation and Evaluation

The primary evaluation employed leave-one-subject-out (LOSO) cross-validation, in which all trials from one pilot were held out as the test set, while the remaining pilots formed the training set [29]. Label construction, feature imputation, feature selection, and classifier fitting were all performed within the training fold, ensuring that no information from the held-out pilot leaked into any stage of the pipeline. LOSO estimates how a classifier would perform when applied to a new, unseen pilot.

Two secondary within-subject analyses were conducted to support comparison with prior subject-dependent studies and to clarify the distinction between personalised and unseen-pilot prediction. First, a subject-dependent window validation split was run separately within each pilot: windows from the same pilot were stratified into training and test folds, and window-level predictions were aggregated to the trial level. This analysis approximates the personalised validation protocols commonly used in physiological computing, although overlapping windows from the same flight segment can appear in both training and test data. Second, a stricter within-pilot leave-one-trial-out analysis held out one flight segment from a pilot and trained on that pilot’s remaining segments. Because each pilot contributed only four flight trials, folds with a single training class were omitted; this analysis is therefore reported as a feasibility and sensitivity check rather than as a primary estimate.

Classification performance was evaluated at the trial level using macro F1 (primary metric), accuracy, and balanced accuracy. Macro F1 serves as the primary metric because it weights both classes equally regardless of class imbalance. Statistical significance was assessed via cluster bootstrap confidence intervals (B=10,000 resamples over pilots) and permutation tests (n=5000). Benjamini–Hochberg FDR-adjusted *p*-values are reported alongside raw permutation *p*-values across all ten model–target cells (α=0.05) [46]. All analyses were conducted with random seed 42.

To confirm that performance did not depend on the a priori hyperparameter choices, we additionally ran nested LOSO cross-validation. Within each outer training fold an inner subject-grouped (GroupKFold) search over a small hyperparameter grid selected the configuration, blind to the held-out pilot; the selected model was then refitted on the full training fold and applied to the held-out pilot. This yields an unbiased estimate of expected performance under honest hyperparameter selection. Results are reported in Section 3.4.

## 3. Results

### 3.1. Primary LOSO Classification Performance

Ten model–target cells (five classifiers × two targets) were evaluated under LOSO cross-validation. Table 3 summarises the results, reporting both raw permutation *p*-values and Benjamini–Hochberg FDR-adjusted *p*-values across all ten cells. For stress, the best-performing classifier was LightGBM on EDA, achieving 61.5% accuracy and macro F1 = 0.611 [95% CI: 0.521, 0.698], significantly exceeding chance ($p_{\mathrm{raw}}=0.008$, $p_{\mathrm{adj}}=0.033$). Linear SVC on combined features ranked second (accuracy = 60.7%, F1 = 0.607, $p_{\mathrm{adj}}=0.033$). Although four of five stress classifiers achieved macro F1 above 0.50, only two survived FDR correction.

**Figure 6:**
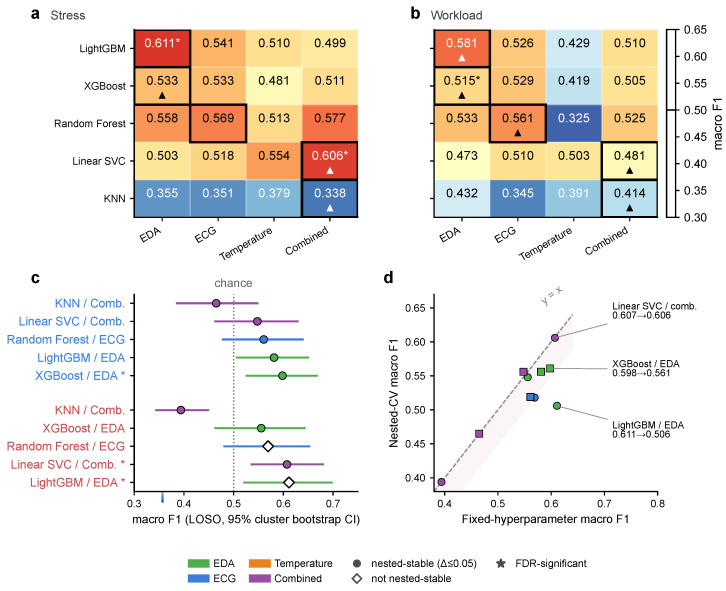
LOSO model-by-modality performance landscape. (**a**,**b**) Macro F1 heatmaps for stress and workload across the five classifiers and four modalities (factorial analysis); bold outlines mark the prespecified configurations, ^*^ marks FDR-significant cells, and ▲ marks nested-stable cells (|fixed − nested| ≤ 0.05). (**c**) Forest plot of the ten prespecified cells with 95% cluster bootstrap confidence intervals (chance = 0.5; ^*^ FDR-significant; filled circle = nested-stable, open diamond = not). (**d**) Fixed-hyperparameter versus nested-CV macro F1 for every cell, with the y=x line.

For workload, XGBoost on EDA achieved the highest performance (accuracy = 60.0%, macro F1 = 0.598 [0.526, 0.668]), significantly exceeding chance after FDR correction ($p_{\mathrm{adj}}=0.033$). LightGBM on EDA ranked second (F1 = 0.581, $p_{\mathrm{raw}}=0.045$, $p_{\mathrm{adj}}=0.113$); this result did not survive FDR correction. Figure 6 summarises the full model-by-modality landscape (panels a,b), the ten prespecified cells with cluster bootstrap CIs (panel c), and the fixed-versus-nested comparison (panel d).

### 3.2. LOSO Versus Within-Subject Validation

Among the prespecified configurations, the two EDA-modality classifiers ranked first and second for both targets. As these were the only configurations pairing gradient-boosted models with a single-modality feature set, the observed advantage may partly reflect model-family differences rather than modality effects alone. Notably, the 102-feature combined set did not consistently outperform single-modality configurations.

**Figure 7:**
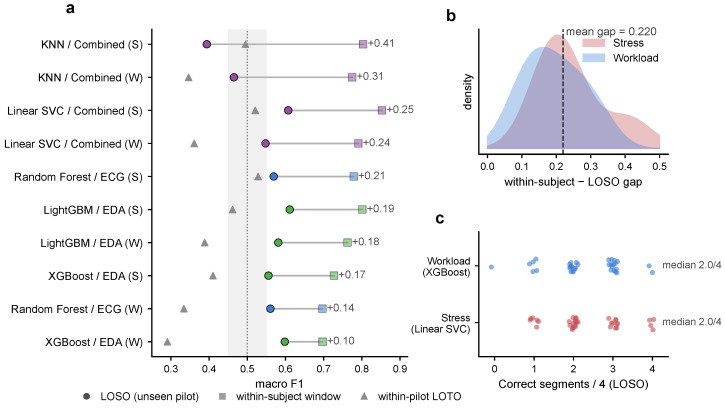
Personalised calibration inflates apparent performance relative to unseen-pilot LOSO validation. (**a**) Dumbbell plot of each model–modality cell (S = stress, warm red; W = workload, blue): LOSO (filled circle), within-subject window (open square), and within-pilot leave-one-trial-out (grey triangle), ordered by the LOSO-to-within gap; the grey band marks the chance region. (**b**) Distribution of the within-subject minus LOSO gap by target (mean gap 0.220). (**c**) Per-pilot LOSO prediction stability for the primary configurations: number of correctly classified segments out of four. Within-subject window validation is optimistic because windows from the same pilot and segment can appear in both training and test folds.

Table 4 compares the primary LOSO results with subject-dependent within-subject window validation. Within-subject window validation produced substantially higher trial-level macro F1 in all ten cells (range: 0.697 to 0.853; mean: 0.769), with an average increase of 0.220 over LOSO. The best within-subject scores were obtained by Linear SVC on combined features for both stress (F1 = 0.853) and workload (F1 = 0.791). These values are more directly comparable with prior subject-dependent studies. However, they should be interpreted as optimistic estimates of personalised performance, because windows from the same pilot and flight segment can appear in both training and test folds. The validation gap, its distribution across cells, and per-pilot LOSO prediction stability are visualised in Figure 7.

A stricter within-pilot leave-one-trial-out sensitivity analysis yielded much lower performance. The best stress result was Random Forest on ECG (macro F1 = 0.529), while the best workload result was LightGBM on EDA (macro F1 = 0.388). This stricter analysis was limited by the study design: each pilot contributed only four analysed flight trials, and folds with single-class training data had to be omitted (18 omitted stress folds and 11 omitted workload folds). The contrast between these two within-subject analyses indicates that high subject-dependent window scores mainly reflect personalised calibration combined with repeated-window and trial-identity information. Reliable within-pilot prediction of a new flight segment would require more repeated trials per pilot.

### 3.3. Full Model × Modality Factorial Analysis

To disentangle the confounding of classifier family with modality in the primary analysis, a full factorial crossing of all five classifiers with all four modalities (EDA, ECG, temperature, combined) was conducted, producing 20 configurations per target evaluated under LOSO (Table 5). For stress, the highest macro F1 was achieved by LightGBM on EDA (0.611), followed by Linear SVC on combined features (0.606) and Random Forest on combined features (0.577). Importantly, the EDA advantage was not universal. Random Forest performed comparably on ECG (0.569) and combined (0.577) as on EDA (0.558), and Linear SVC performed best on combined features (0.606) rather than EDA (0.503). For workload, LightGBM on EDA (0.581) and Random Forest on ECG (0.561) were the top performers. Temperature was consistently the weakest standalone modality (best: Linear SVC/stress, F1 = 0.554; worst: Random Forest/workload, F1 = 0.325). These factorial results indicate that the apparent EDA dominance in the primary analysis was partly attributable to the pairing of EDA with gradient-boosted models. When all classifiers were evaluated across all modalities, the modality effect was weaker and more model-dependent than the primary analysis suggested.

### 3.4. Robustness: Nested Cross-Validation, Feature Count, and Selection Stability

Three analyses test the robustness of the primary results to hyperparameter choice and feature dimensionality.

Nested cross-validation. Re-evaluating all ten model–target cells under nested LOSO with an inner subject-grouped hyperparameter search changed macro F1 by only −0.026 on average (8 of 10 cells within 0.05). The two foregrounded configurations were stable (stress Linear SVC 0.607 to 0.606; workload XGBoost 0.598 to 0.561), whereas the single highest stress estimate, LightGBM on EDA, fell from 0.611 to 0.506; we therefore adopt the hyperparameter-insensitive Linear SVC as the primary stress configuration. No single hyperparameter setting dominated across folds, indicating that at this sample size any one fixed configuration is a noisy point estimate (Table 6, panels A and B).

Feature count. For the EDA classifier, trial-level macro F1 peaked at the selected 10 features (stress 0.611, workload 0.581) and degraded as more were admitted (using all 28 features lowered stress macro F1 to 0.444), so performance does not improve with dimensionality.

**Figure 8:**
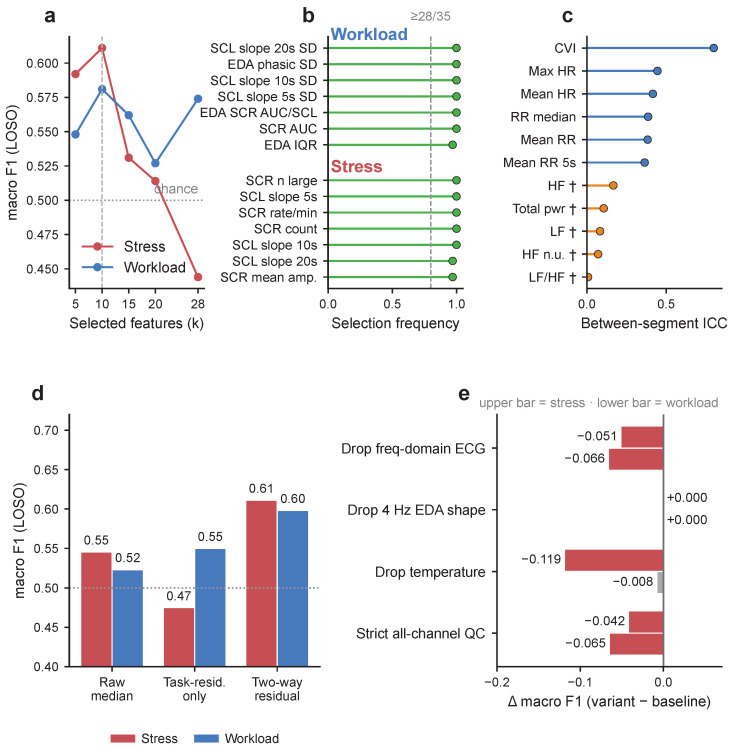
Robustness to feature count, label definition, signal reliability, and quality-control choices. (**a**) Trial-level LOSO macro F1 of the EDA classifier versus the number of selected features *k*; performance peaks at the selected k=10 and degrades when more features are admitted. (**b**) Feature-selection stability: fraction of the 35 LOSO folds in which each top feature was selected. (**c**) Between-segment intraclass correlation (ICC) of cardiac features; frequency-domain indices (†) are near zero on the 56–158 s segments, whereas time-domain indices are far more reliable. (**d**) Label-definition sensitivity: LOSO macro F1 under raw median split, task-residual-only, and two-way residual labels. (**e**) QC and ablation deltas (Δ macro F1) for the primary configurations (independent analyses; upper bar stress, lower bar workload).

Selection stability. Across the 35 LOSO folds, the selected feature sets were highly stable (for stress, all ten top features were selected in at least 28 of the 35 folds; for workload, eight of ten). The primary models thus operate on a small, stable set of roughly 10 features rather than the full 102-feature space, mitigating the overfitting concern that the raw feature-to-sample ratio might suggest. Figure 8 shows the feature-count curve, the feature-selection stability across folds, the ultra-short HRV reliability, the label-definition sensitivity, and the QC/ablation deltas.

### 3.5. Label Sensitivity Analysis

Three labelling approaches were compared for the best model–modality configurations under LOSO: fold-safe two-way residual (primary), task-residual only (removing only the task mean), and raw median split. The fold-safe approach yielded the highest macro F1 for both targets (stress: 0.611 vs. 0.475 and 0.545; workload: 0.598 vs. 0.550 and 0.523). The task-residual-only approach, which does not remove pilot-mean differences, performed worst for stress, presumably because inter-pilot scaling heterogeneity obscures the physiological signal. These results suggest that the two-way residual labelling approach provides a classification target that is more amenable to learning from physiological features than alternative label definitions (Figure 8d).

### 3.6. Feature Importance Analysis

**Figure 9:**
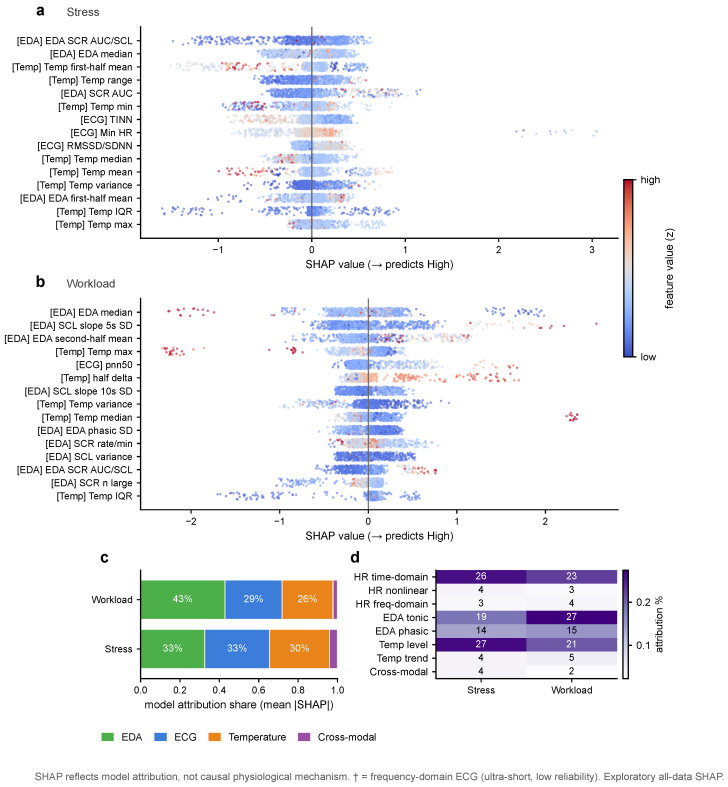
Directional physiological signatures of workload and stress from SHAP values (exploratory all-data SHAP; LightGBM on combined features). (**a**,**b**) SHAP beeswarm for stress and workload: each point is one window, positioned by its SHAP value (right = pushes towards high) and coloured by the within-participant *z*-scored feature value; modality tags precede each feature and † marks low-reliability frequency-domain ECG features. (**c**) Modality-level model-attribution share (mean |SHAP|). (**d**) Feature-category attribution contrast between stress and workload. SHAP reflects model attribution, not causal physiological mechanism.

To identify which features drove classification decisions, SHAP (SHapley Additive exPlanations) values [47] were computed via TreeExplainer on a LightGBM model trained on all available data with the combined feature set. Because these are all-data rather than out-of-fold values, they are interpreted as exploratory and reflect model attribution rather than causal physiology. Figure 9 displays the top 15 features by mean absolute SHAP value for each target, with directionality and feature-value colouring.

For stress (Figure 9a), the most influential ECG features were time-domain metrics (heart rate mean, SDNN) and Poincaré descriptors, consistent with the known sensitivity of vagal-withdrawal indices to cognitive-affective demands. EDA importance was dominated by phasic metrics (SCR rate, SCR amplitude, short-window SCL slope), confirming the relevance of phasic sympathetic activation to stress detection. Temperature features were led by absolute level descriptors rather than variability metrics, suggesting that thermoregulatory shifts across the flight contributed more to classification than within-window fluctuations. In the combined model, top features spanned all three modalities.

For workload (Figure 9b), ECG features emphasised heart rate extrema and the LF/HF ratio, consistent with sympatho-vagal rebalancing during high-demand segments. The prominence of LF/HF should, however, be read with caution: as reported in the Materials and Methods section and in Section 3.4, LF/HF has near-zero between-segment reliability (ICC = 0.007) on our 56–158 s segments, so its SHAP ranking is treated as exploratory rather than as evidence of a robust frequency-domain effect; the heart-rate (time-domain) features are the more reliable contributors. EDA importance was dominated by tonic-level descriptors (SCL variance, first-half mean, phasic standard deviation) rather than the phasic SCR rate that dominated stress, suggesting that workload engages more sustained autonomic arousal than the transient sympathetic bursts characteristic of stress responses. Temperature importance was concentrated in absolute extrema and temporal trend features.

### 3.7. Summary by Research Question

RQ1: Three of ten cells significantly exceeded chance after FDR correction (α=0.05): stress LightGBM/EDA (F1 = 0.611), stress Linear SVC/combined (F1 = 0.607), and workload XGBoost/EDA (F1 = 0.598), all $p_{\mathrm{adj}}=0.033$. Of these, the nested-stable stress Linear SVC/combined and workload XGBoost/EDA are foregrounded as the primary configurations (Section 3.4). RQ2: Subject-dependent within-subject window validation produced a substantially higher macro F1 than LOSO (mean gap = 0.220), but stricter within-pilot leave-one-trial-out validation was unstable with only four trials per pilot. RQ3: EDA-based gradient-boosted models achieved the highest F1 for both targets under LOSO, but the factorial analysis showed this advantage is partly model-dependent; temperature was consistently the weakest modality.

## 4. Discussion

### 4.1. Principal Findings

Three principal findings emerged. First, consumer-grade wearable signals exhibit weak but statistically detectable correlates of task-relative stress deviations under strict subject-disjoint validation. The best stress classifier (LightGBM on EDA) achieved a macro F1 = 0.611 [95% CI: 0.521, 0.698], significantly exceeding chance after FDR correction ($p_{\mathrm{adj}}=0.033$). This result suggests that EDA signals recorded by the Empatica Embrace Plus during real flight contain individual-level information correlated with stress deviation labels. However, the modest performance and wide confidence interval indicate exploratory rather than operationally reliable detection.

Second, workload classification proved more challenging. The best model (XGBoost on EDA, F1 = 0.598 [0.526, 0.668]) also significantly exceeded chance ($p_{\mathrm{adj}}=0.033$) but with a narrower margin. This asymmetry may reflect the stronger association between task structure and workload ratings. Even after two-way residual labelling, the systematic increase in workload from Takeoff through Landing leaves less within-pilot residual variance for physiological models to exploit.

Third, subject-dependent within-subject window validation produced much higher apparent performance than LOSO, with best trial-level macro F1 values of 0.853 for stress and 0.791 for workload. These results are useful for comparison with prior personalised validation studies, but they do not estimate generalisation to unseen pilots. The stricter within-pilot leave-one-trial-out analysis performed poorly and omitted folds with single-class training data, indicating that four trials per pilot are insufficient for stable personalised prediction of new flight segments.

### 4.2. Comparison with Prior Studies

Table 7 summarises selected pilot workload and stress studies that used at least one of the peripheral channels evaluated here, namely, ECG, EDA, or skin temperature. The comparison is broader than a strict same-sensor comparison because most aviation studies combine these signals with EEG, respiration, EMG, pupil measures, or other channels. For breadth, and following the reviewers’ suggestion, the table now also includes representative real-flight EEG and wearable-ECG studies [23,24]. Other EEG- and fNIRS-headset studies are cited for context [22,48,49] but, given their markedly different sensing burden, are not used to rank performance. The table should therefore be read as a methodological map rather than a ranking of model quality.

Prior workload studies have largely relied on ECG-derived features. They range from an in-flight helicopter study with two test pilots and an ECG-based SVM regressor [50] to simulator studies using ECG-derived classifiers in small or moderate pilot samples [51,52]. More recent simulator work has integrated ECG-derived features with EDA and pupil diameter, reporting relatively high AUC and accuracy values under task demand labels [53]. Pilot stress prediction has been studied less often with the same peripheral channels. Li et al. [54] reported high accuracy for two-, three-, and four-class pilot stress detection using ECG, EDA, skin temperature, EMG, and respiration. Johannes et al. [55] used ECG, EDA, and finger temperature to characterise psychophysiological activation during challenging real and simulated manoeuvres. These studies support the relevance of the present signal set, but they also show that published performance is strongly conditioned by task definition, sensor mix, and validation design [25,56]. A recent example is Qin et al. [45], who reported real-time pilot-stress recognition in naturalistic scenarios using pulse-interval signals and a hybrid SVM-LSTM model. Their approach is closely related in aim, but, like most of the studies in Table 7, it is evaluated in a within-subject setting; the contrast with our subject-disjoint LOSO estimates again illustrates that the validation protocol, more than the sensor or model family, governs the reported performance level.

The present within-subject window results are numerically closer to the subject-dependent or simulator-based literature, with macro F1 values of 0.85 for stress and 0.79 for workload. These values are most compatible with personalised retrospective classification after calibration, where data from the same pilot help define the model. The primary LOSO results are lower (macro F1 of 0.61 for stress and 0.60 for workload), but they answer a stricter question: whether consumer-grade ECG, EDA, and skin temperature features generalise to a pilot unseen during training. The comparison indicates that the present study should not be framed as matching the highest simulator accuracies. A more cautious interpretation is that it quantifies the cross-pilot generalisation boundary in actual Cessna 172 training flights, using fold-safe residual labels that reduce pilot scale-use and task-identity confounding.

**Table 7:** Selected comparison studies using ECG, EDA, or skin temperature for pilot workload, stress, or closely related autonomic state inference. WL = workload, W subj = within-subject, CV = cross-validation, PAV = psychophysiological activation value, cl = classes.

Study	*N*	Setting	Signals	Valid.	Target	Perf.
Present study, real flight, ECG + EDA + temperature, residual labels						
Present study	35	Real flight	ECG, EDA, Temp	W subj	Stress (2 cl)	F1 0.85
Present study	35	Real flight	ECG, EDA, Temp	W subj	WL (2 cl)	F1 0.79
Present study	35	Real flight	ECG, EDA, Temp	LOSO	Stress (2 cl)	F1 0.61
Present study	35	Real flight	ECG, EDA, Temp	LOSO	WL (2 cl)	F1 0.60
Prior pilot studies using at least one matching peripheral channel						
Ghosh Hajra et al. [50]	2	Real helicopter	ECG, EEG	CV	WL difficulty (3 levels)	MSE 0.17
Mohanavelu et al. [51]	7	Simulator	ECG	CV	WL (2 cl)	Acc 0.72 to 0.76
Han et al. [16]	8	Simulator	EEG, ECG, EDA, Resp	W subj	Pilot mental states	Acc 0.85
Li et al. [54]	14	Simulator	ECG, EDA, Temp, EMG, Resp	W subj	Stress (2 to 4 cl)	Acc 0.85 to 0.93
Wang et al. [53]	19	Simulator	ECG, EDA, pupil	Split	WL (3 levels)	Acc 0.82 to 0.88
Vindigni et al. [52]	34	Simulator	ECG	CV	WL (2 cl)	Acc 0.83
Johannes et al. [55]	15	Real + sim	ECG, EDA, Temp	Task contrast	Arousal during manoeuvres	PAV, no ML
Real-flight studies using EEG or wearable ECG (added for breadth)						
Taheri Gorji et al. [23]	10	Real flight	EEG	10-fold CV	WL (3 levels)	Acc 0.92
Guo et al. [24]	90	Real flight	ECG (HRV)	CV	Fatigue (3 levels)	Acc 0.89

### 4.3. Methodological Implications

The mean 0.220 gap between subject-dependent within-subject window validation and LOSO is consistent with the well-documented performance difference between personalised and subject-disjoint evaluation in physiological computing. Comparable gaps have been reported in multimodal affective computing [17], and high within-driver accuracy in driving-stress paradigms has not been replicated under subject-disjoint validation [19]. When windows from the same pilot and flight segment appear in both training and test folds, the classifier can learn pilot-specific patterns (e.g., resting heart rate, baseline skin conductance). Such trial-specific signatures do not reflect generalisable state discrimination. For deployment on unseen pilots, LOSO provides the appropriate estimate. Within-subject validation remains informative, but it answers a different question: whether a personalised model may be useful after collecting calibration data from the same pilot.

The stricter within-pilot leave-one-trial-out analysis tempers the apparently strong within-subject window results. With only four analysed flight trials per pilot, many folds had insufficient class variation for training, and performance dropped sharply. A practically useful personalised model would therefore require more repeated flights or more labelled task segments per pilot. The present design is adequate for estimating between-pilot generalisation under LOSO and for illustrating the upper bound of subject-dependent window validation. It is not, however, sufficient to establish stable personalised forecasting of a new segment within the same pilot.

The fold-safe two-way residual labelling addresses two confounds: inter-pilot scale-use differences [6,7] and task-identity leakage under fixed ordering. Subtracting each pilot’s mean score removes between-pilot scaling heterogeneity; subtracting the training-set task mean reduces the association between labels and task identity. The classification target is therefore not raw workload or stress but the binary sign of a residual, that is, whether a pilot’s response exceeded what the task typically elicits relative to that pilot’s own baseline.

EDA is broadly sensitive to sympathetic arousal [11], but sympathetic activation is not specific to stress; it may also reflect motor effort, thermoregulation, novelty, or instructor interaction. Similarly, mental workload is a multidimensional construct rather than a unitary quantity mapping directly onto a binary physiological threshold [3]. The above-chance performance therefore reflects a composite of stress-related activation and unmeasured confounds, and should not be interpreted as evidence that EDA selectively tracks these constructs. A plausible pathway runs through the cognitive-appraisal framework [18]. When a pilot perceives a segment as more demanding than expected, sympathetic activation elevates eccrine activity beyond the task-level norm, precisely the deviation that the residual label is designed to capture. However, this pathway is not specific, and the above-chance classification may reflect both stress-related sympathetic activation and unmeasured confounds sharing variance with the residual label. Future work should evaluate convergent validity against independent markers (e.g., instructor ratings, performance metrics, full NASA-TLX).

### 4.4. Modality Observations

In the primary analysis, EDA-based gradient-boosted models achieved the highest classification performance for both targets. Tonic skin-conductance level and phasic SCR metrics are well-established correlates of sympathetic arousal [11]. The Embrace Plus wristband captured sufficient EDA signal quality during real flight for trial-level classification, consistent with findings from driving and laboratory paradigms [17,19]. However, the full factorial analysis revealed that this advantage is partly model-dependent. EDA was the best modality for LightGBM (stress F1 = 0.611) but not for Random Forest (best on ECG, F1 = 0.569) or Linear SVC (best on combined, F1 = 0.606). The EDA–gradient-boosting synergy may reflect the capacity of tree ensembles to exploit nonlinear SCR features that linear or distance-based classifiers cannot leverage as effectively.

Random Forest on ECG-derived features achieved moderate performance (stress F1 = 0.569, workload F1 = 0.561), ranking third for both targets. The shorter segment durations (56–158 s) may limit the reliability of frequency-domain and nonlinear ECG-derived features that typically require longer recording windows [8]. Time-domain features (mean RR, SDNN, RMSSD) are more robust to short windows and likely drive the ECG model’s performance.

The 102-feature combined set did not consistently outperform single-modality EDA. This may reflect increased dimensionality without proportional signal gain, collinearity between modalities sharing sympathetic-activation variance, and the regularisation challenge of fitting more features to a small training set under LOSO. Multimodal fusion may therefore require careful feature engineering or dimensionality reduction to yield consistent benefit in small-sample operational settings. Temperature was consistently the weakest standalone modality (best: Linear SVC/stress, F1 = 0.554; worst: Random Forest/workload, F1 = 0.325). This likely reflects both the slow thermal response dynamics relative to segment durations and environmental confounds (e.g., airflow, cockpit temperature) that introduce noise uncorrelated with self-reported states. It is, however, not redundant in the multimodal set: removing it lowered combined performance (e.g., Linear SVC/stress from 0.607 to 0.488), although its absolute-level features track flight time almost monotonically and so act partly as a slow time-on-task proxy. We therefore retain temperature but flag its environmental sensitivity and recommend logging ambient cabin temperature in future work.

### 4.5. Practical Implications

The most plausible near-term application is supporting structured post-flight debriefing, which improves trainee performance by approximately 25% across aviation and related high-stakes domains [57,58]. A practical workflow would involve: (a) physiological data collected passively during flight; (b) offline analysis to identify segments where a pilot’s deviation label indicates higher-than-expected workload or stress; (c) flagged segments presented to the instructor as discussion prompts rather than diagnostic assessments; and (d) instructor judgment remaining the primary basis for training decisions. No classifier output should be used for grading, disciplinary action, or licensing decisions.

At macro F1 ≈ 0.60, the classifier produces substantial false positives and false negatives. At the best stress classifier’s operating point (recall = 0.70, specificity = 0.53), a typical four-segment flight would yield approximately two flagged segments, of which one is a true positive and one a false positive. In the debriefing context, false positives constitute tolerable prompts that the instructor can readily dismiss. False negatives represent missed opportunities but do not compromise safety, as the system augments rather than replaces instructor judgement. Real-time in-flight intervention is not supported at these error rates.

Hardware costs are modest (Polar H10 ∼$90; Embrace Plus ∼$300). Deployment, however, requires data-management infrastructure, instructor training, and governance safeguards, including informed consent, access controls, explicit prohibition of use in licensing decisions, and data retention policies. Regulatory consultation would be necessary to determine whether physiological monitoring in training falls under existing aviation-authority oversight.

### 4.6. Limitations

All pilots completed segments in a fixed order (Takeoff → Steep Turn → Stall → Landing), precluding separation of task effects from time-on-task, fatigue, and physiological drift. The two-way residual mitigates but does not eliminate this confound; counterbalanced designs would be required. To quantify the residual confound, we regressed features and labels on segment order within each pilot. Order accounted for a modest share of within-pilot feature variance (median R-squared about 0.16–0.19 across modalities). The two-way residual roughly halved the order-driven variance of the stress label (within-pilot R-squared from 0.33 to 0.115), with a smaller reduction for workload (0.20 to 0.199). This asymmetry is consistent with workload being more tightly coupled to task structure. The residualisation therefore reduces, but does not remove, the ordering confound, and a counterbalanced protocol remains necessary for causal separation. The cohort of 35 pilots with 135 trials is typical for operationally embedded human factors research but small by machine learning standards. The LOSO evaluation becomes particularly conservative with small samples, and the wide confidence intervals (e.g., [0.521, 0.698]) underscore the preliminary nature of these estimates.

Single-item 0–10 ratings impose a reliability ceiling and may not capture the multidimensional nature of workload and stress [5,59]. The two-class framing discards continuous information in self-report scores; future work could explore ordinal classification if finer resolution is desired. Real-flight data introduce uncontrolled variables (weather, traffic, instructor interaction, cockpit temperature) that may affect physiology independently of self-reported states. The primary analysis paired each classifier with a specific feature set; although the factorial analysis addressed this limitation, the factorial runs used default hyperparameters rather than per-cell tuning. Both the fold-safe residual labels and the within-participant normalisation are session-retrospective, suitable for post-flight analysis but requiring adaptation for real-time deployment. Motion artefacts during active manoeuvres were handled by automated quality flagging, masking, and fold-internal imputation rather than by a dedicated motion-artefact-removal algorithm. A stricter all-channel-usable criterion left the conclusions unchanged in direction (stress 0.611 to 0.569; workload 0.598 to 0.533). An acceleration-informed correction stage would nonetheless be preferable and is a priority for the real-time extension. Several factors known to influence electrodermal and cardiac measures (caffeine and nicotine intake, sleep, medication, and post-flight psychological state) were not systematically recorded under this operational protocol and should be logged in future studies. All flights used a Cessna 172 under VFR [31]; generalisation to other airframes, IFR, or multi-crew operations remains to be demonstrated.

Three priority follow-up directions are independent cohort validation at different training units or aircraft types, counterbalanced task ordering with ambient-thermal monitoring, and longitudinal multi-flight monitoring to enable individualised baseline calibration.

## 5. Conclusions

Physiological signals from consumer-grade wearable sensors recorded during real Cessna 172 flight training show weak but statistically detectable associations with pilots’ task-relative workload and stress deviations. Under LOSO cross-validation with fold-safe two-way residual labels and FDR correction, three of ten model–target cells significantly exceeded chance, stress LightGBM/EDA (F1 = 0.611), stress Linear SVC/combined (F1 = 0.607), and workload XGBoost/EDA (F1 = 0.598), all $p_{\mathrm{adj}}=0.033$. Nested cross-validation confirmed the stress Linear SVC and workload XGBoost configurations as stable, and these are foregrounded as the primary results. Subject-dependent within-subject window validation produced higher apparent performance (best F1 = 0.853 for stress and 0.791 for workload), but stricter within-pilot leave-one-trial-out validation was unstable and substantially weaker. This pattern confirms that personalised validation and unseen-pilot generalisation represent distinct application scenarios.

Among the tested configurations, EDA-based gradient-boosted models achieved the highest fixed-hyperparameter performance for both targets. Under nested cross-validation, however, the gradient-boosted advantage for stress did not persist, and the hyperparameter-insensitive Linear SVC on combined features is foregrounded as the primary stress configuration. A supplementary full factorial analysis crossing all five classifiers with all four modalities confirmed that this advantage is partly model-dependent. EDA excelled with gradient-boosted classifiers, whereas ECG and combined features matched or exceeded EDA performance under other model families. The fold-safe two-way residual labelling framework removes both inter-pilot scale-use differences and task-level mean effects. The resulting classification target is aligned with the question of whether a pilot experienced higher-than-expected workload or stress on a given task, a framing most applicable to post-flight debriefing rather than real-time monitoring.

Together, these findings provide preliminary evidence that consumer-grade wearable signals encode detectable physiological correlates of self-reported workload and stress deviations, with potential relevance to post-flight debriefing applications. The modest LOSO performance levels (macro F1 ≈ 0.60) and wide confidence intervals indicate that current systems should be regarded as exploratory screening tools rather than operationally reliable diagnostic instruments for new pilots. Future work should pursue independent cohort validation, counterbalanced task ordering, and longitudinal multi-flight monitoring to strengthen both cross-pilot and personalised modelling.

## Figures and Tables

**Table 1:** Cohort and protocol summary.

Field	Value
Parent cohort [31]	N=35 pilots (mixed PPL/CPL/SPL/SPP)
Analysis segments	Segments 2–5 (Takeoff, Steep Turn, Stall, Landing); Baseline excluded
Trial-level observations	135 (after label construction)
Label balance (high:low; stress/workload)	66:69/62:73
Aircraft/regime	Cessna 172, VFR, certified flight instructor present
Self-report	0–10 Likert (verbal anchors at 0, 5, 10) for stress + workload
Sensors	Polar H10 (ECG); Empatica Embrace Plus (EDA, 4 Hz; temperature)

**Table 2:** Participant characteristics (N=35). Flight hours are summarised by median, interquartile range, and range owing to their skewed distribution.

Characteristic	Value
Age, years—mean ± SD (range)	29.3 ± 16.3 (18–78)
Sex—*n* (male/female)	31/4
Licence—*n*	PPL 20, CPL 10, SPL 2, SPP 2 (1 unspecified)
Flight hours—median [IQR] (range)	160 [104–322] (20–1800)
Additional rating (IFR/Multi/Night)—*n*	23
Pre-flight stress (0–10)—mean ± SD	1.9 ± 1.3

**Table 3:** LOSO classification performance (N=135 trials, 35 pilots). Ranked by macro F1. $p_{\mathrm{raw}}$: permutation test; $p_{\mathrm{adj}}$: Benjamini–Hochberg FDR-adjusted across all ten cells. Significance (based on $p_{\mathrm{adj}}$): ^†^ $p_{\mathrm{adj}}<0.05$.

Target	Model (Modality)	Acc	F1	F1 [95% CI]	$p_{\mathrm{raw}}$	$p_{\mathrm{adj}}$
Stress	LightGBM (EDA)	0.615	0.611	[0.521, 0.698]	0.008	0.033 ^†^
Stress	Linear SVC (Comb.)	0.607	0.607	[0.536, 0.680]	0.010	0.033 ^†^
Stress	Random Forest (ECG)	0.570	0.569	[0.481, 0.652]	0.068	0.113
Stress	XGBoost (EDA)	0.556	0.556	[0.463, 0.643]	0.128	0.160
Stress	KNN (Comb.)	0.526	0.394	[0.344, 0.449]	0.310	0.310
Workload	XGBoost (EDA)	0.600	0.598	[0.526, 0.668]	0.008	0.033 ^†^
Workload	LightGBM (EDA)	0.585	0.581	[0.507, 0.650]	0.045	0.113
Workload	Random Forest (ECG)	0.563	0.561	[0.478, 0.639]	0.109	0.156
Workload	KNN (Comb.)	0.578	0.465	[0.386, 0.548]	0.063	0.113
Workload	Linear SVC (Comb.)	0.548	0.548	[0.463, 0.629]	0.156	0.173

**Table 4:** LOSO versus subject-dependent within-subject window validation, trial-level macro F1. Gap = within-subject − LOSO. The within-subject analysis is included for comparison with prior personalised validation studies and should not be interpreted as unseen-pilot generalisation.

Target	Model (Modality)	LOSO F1	Within F1	Gap	Within *N*
Stress	Linear SVC (Comb.)	0.607	0.853	+0.246	132
Stress	KNN (Comb.)	0.394	0.804	+0.410	132
Stress	LightGBM (EDA)	0.611	0.801	+0.190	132
Stress	Random Forest (ECG)	0.569	0.779	+0.210	132
Stress	XGBoost (EDA)	0.556	0.727	+0.172	132
Workload	Linear SVC (Comb.)	0.548	0.791	+0.243	134
Workload	KNN (Comb.)	0.465	0.775	+0.310	134
Workload	LightGBM (EDA)	0.581	0.763	+0.181	134
Workload	XGBoost (EDA)	0.598	0.697	+0.099	134
Workload	Random Forest (ECG)	0.561	0.697	+0.136	132
Mean		0.549	0.769	+0.220	133

**Table 5:** Full factorial LOSO macro F1 (5 classifiers × 4 modalities × 2 targets). Bold: best modality per row.

	Stress				Workload			
Model	EDA	ECG	Temp	Comb	EDA	ECG	Temp	Comb
LightGBM	**0.611**	0.541	0.510	0.499	**0.581**	0.526	0.429	0.510
XGBoost	0.533	0.533	0.481	0.511	0.515	**0.529**	0.419	0.505
Random Forest	0.558	0.569	0.513	**0.577**	0.533	**0.561**	0.325	0.525
Linear SVC	0.503	0.518	0.554	**0.606**	0.473	**0.510**	0.503	0.481
KNN	0.355	0.351	**0.379**	0.338	**0.432**	0.345	0.391	0.414

**Table 6:** Robustness analyses. (**A**) Nested LOSO cross-validation with an inner subject-grouped hyperparameter search, versus the fixed-hyperparameter results, for all ten model–target cells. (**B**) Sensitivity of the primary configurations to feature and quality-control choices. Δ is variant minus baseline macro F1.

**(A) Nested vs. fixed-hyperparameter macro F1**				
**Target**	**Model (Modality)**	**Fixed**	**Nested**	Δ
Stress	Linear SVC (Comb.)	0.607	0.606	−0.001
Stress	LightGBM (EDA)	0.611	0.506	−0.106
Stress	XGBoost (EDA)	0.556	0.548	−0.007
Stress	Random Forest (ECG)	0.569	0.518	−0.052
Stress	KNN (Comb.)	0.394	0.394	0.000
Workload	XGBoost (EDA)	0.598	0.561	−0.037
Workload	LightGBM (EDA)	0.581	0.556	−0.025
Workload	Linear SVC (Comb.)	0.548	0.556	+0.008
Workload	Random Forest (ECG)	0.561	0.519	−0.042
Workload	KNN (Comb.)	0.465	0.465	0.000
**(B) Sensitivity of primary configurations**				
**Analysis**	**Target (Model)**	**Base**	**Variant**	Δ
Drop frequency-domain ECG	Stress (RF/ECG)	0.569	0.518	−0.051
Drop frequency-domain ECG	Workload (RF/ECG)	0.561	0.495	−0.066
Drop 4 Hz shape features	Stress (LGBM/EDA)	0.611	0.611	0.000
Drop 4 Hz shape features	Workload (XGB/EDA)	0.581	0.581	0.000
Drop temperature (Comb.)	Stress (SVC)	0.607	0.488	−0.119
Drop temperature (Comb.)	Workload (SVC)	0.548	0.540	−0.008
Strict all-channel QC	Stress (LGBM/EDA)	0.611	0.569	−0.042
Strict all-channel QC	Workload (XGB/EDA)	0.598	0.533	−0.065

## Data Availability

The analysis code, feature-generation scripts, and cross-validation result tables are available from the corresponding author upon reasonable request.

## Abbreviations

The following abbreviations are used in this manuscript:

ANOVA	Analysis of variance
AUC	Area under the curve
CI	Confidence interval
CPL	Commercial Pilot Licence
ECG	Electrocardiogram
EDA	Electrodermal activity
EEG	Electroencephalogram
FDR	False discovery rate
fNIRS	Functional near-infrared spectroscopy
HF	High-frequency HRV power
HRV	Heart rate variability
ICC	Intraclass correlation coefficient
IQR	Interquartile range
LF	Low-frequency HRV power
LOSO	Leave-one-subject-out
MAD	Median absolute deviation
NASA-TLX	NASA Task Load Index
PPL	Private Pilot Licence
QC	Quality control
RMSSD	Root mean square of successive RR differences
ROC	Receiver operating characteristic
SCL	Skin conductance level
SCR	Skin conductance response
SD	Standard deviation
SDNN	Standard deviation of NN intervals
SHAP	SHapley Additive exPlanations
SPL	Student Pilot Licence
SPP	Sport Pilot Permit
VFR	Visual flight rules
